# Crystal engineering, crystals and crystallography

**DOI:** 10.1107/S2052252518015014

**Published:** 2018-10-26

**Authors:** Gautam R. Desiraju

**Affiliations:** a Solid State and Structural Chemistry Unit, Indian Institute of Science, Bangalore 560 012, India

**Keywords:** crystal engineering, crystals, crystallography

## Abstract

Crystal engineering is moving into new domains with reducing distance and time scales and new ways of thinking about crystals and crystallography. Its intersections with other subjects are varied and numerous.

Crystal engineering is concerned with molecular crystals and their systematic design. Over the last few years, basically during the period of existence of **IUCrJ**, there has been a huge increase in the growth and impact of this subject with ever increasing applications and products that are likely to improve the quality of life for society at large. **IUCrJ** attempts to capture the best papers in this area, especially those which make fundamental advances as to what one means by terms like ‘molecule’, ‘crystal’ and ‘crystallography’. By expanding the definition of the word ‘molecule’, one can extend the concept of molecularity from three-dimensional to two- and one-dimensional so that metal–organic framework compounds (MOFs) and covalent organic framework compounds (COFs) are also properly included within the scope of crystal engineering. A ‘crystal’ is an ordered, periodic array, but today the term comfortably incorporates varying degrees of order too. Consideration of structures in which the periodicity is less than ideal leads one eventually to amorphous solids and these are actually being studied actively today in the pharmaceutical industry. And what of ‘crystallography’? There are papers in other sections of this journal where one does not even deal with ‘crystals’, however loosely one might define this term. Will crystal engineering move into such territory? In truth, the entire gamut of research activity in the structural sciences is undergoing something of a revolution and crystal engineering is surely not going to be unaffected by these radical changes.

Any growing field needs to be linked with properties or applications of wide general utility. Historically, areas of chemistry that appeared interesting faded with time in the absence of such an impetus. In this regard, crystal engineering has been particularly fortunate in that it has become linked to two predominant applications: gas sorption (for MOFs) and pharmaceutical cocrystals (for organics). Newer applications include mechanical properties, bulk synthesis, biomaterials, catalysis and electronic devices for solar energy conversion. Papers that deal with such applications are already seen in **IUCrJ**.

The 20 or so papers that have been published in **IUCrJ** in 2018 deal with a wide variety of frontier topics and, for representative purposes only, one might mention papers that have dealt with novel intermolecular interactions of the weakest sort (Shukla *et al.*, 2018 ▸), H/D exchange as a tool to control polymorphism (Falk *et al.*, 2018 ▸), the ability of ‘soft’ interactions to direct crystal packing (Sinnwell *et al.*, 2018 ▸), the synthesis of multi-component crystals with large numbers of constituent molecules (Dandela *et al.*, 2018 ▸) and the relationship between crystal chemistry principles and quantum chemistry data (Levi *et al.*, 2018 ▸). The 20 crystal engineering papers appear among the 75 or so that have been published in **IUCrJ** this year, and from a total of the 375 or so that have been published overall since the inception of the journal itself. We are certainly being selective about what we publish, and this is revealed in the impressive impact factor of the journal today. Increasing quantity with quality is one of the more difficult endeavours in any scientific enterprise but I am confident that authors, referees and editors will rise to the occasion and maintain **IUCrJ** as a premier location for papers in crystal engineering as it moves forward into the future.
